# Structural Modelling of Krüppel‐Like Factor 15 Zinc Finger Binding Domain to DNA Using AlphaFold 3.0: Potential Therapeutic Target for Type 2 Diabetes

**DOI:** 10.1111/jcmm.70565

**Published:** 2025-05-24

**Authors:** Anwar Mohammad, Jehad Abubaker, Sulaiman K. Marafie, Eman AlShawaf, Hamad Ali, Fahd Al‐Mulla

**Affiliations:** ^1^ Department of Biochemistry and Molecular Biology Dasman Diabetes Institute Kuwait City Kuwait; ^2^ Department of Medical Laboratory Sciences Faculty of Allied Health Sciences, Health Sciences Center (HSC), Kuwait University Kuwait City Kuwait; ^3^ Department of Genetics and Bioinformatics Dasman Diabetes Institute Kuwait City Kuwait; ^4^ Department of Translation Research Dasman Diabetes Institute Kuwait City Kuwait

**Keywords:** AlphaFold 3.0, computational biology, Krüppel‐like factor 15, metabolic syndrome, type 2 diabetes

## Abstract

Krüppel‐like factor 15 (KLF15) is a transcription factor contributing to the pathophysiology of multiple diseases, including metabolic syndromes. It is 416 residues long, with a C2H2‐type zinc finger (ZnF) domain that binds to GC‐rich regions regulating transcription. The role of KLF15 in glucogenesis and glucose level maintenance is well established. However, the DNA interaction mechanism at the atomic level remains unresolved. Here, we utilised computational structural biology tools to address this knowledge gap. The KLF15 ZnF–domain interacting with DNA was modelled with AlphaFold 3.0. Alanine substitution of the KLF15 ZnF domain–DNA complex revealed that residues K334A, R334A, Y332A and R392A significantly affect the binding affinities (ΔΔG) to DNA. To understand the conformational stability and dynamics of the KLF15 ZnF–domain complexes, 100‐ns molecular dynamics simulations were performed. Additionally, molecular mechanics‐generalised Born (MM/GBSA) surface area was utilised to calculate total binding energies. The binding energies of the wild‐type KLF15 ZnF domain (−94.0 ± 0.17 kcal/mol) demonstrated a more robust binding affinity to DNA than K334A (−30.4 ± 0.35 kcal/mol), R344A (−42.8 ± 0.37 kcal/mol), Y332A (−47.7 ± 0.42 kcal/mol) and R392A (−30.8 ± 0.30 kcal/mol). The findings highlighted the unstable dynamics of the alinine substituted resdiues that consequently reduce the binding free energy compared to the wild‐type KLF15 ZnF domain. In conclusion, the four identified residues are essential to recognise KLF15 ZnF DNA binding and can be considered potential hotspots for the therapeutics development for type 2 diabetes.

## 1 Introduction

Krüppel‐like factors (KLFs) are transcription factors expressed in the liver, adipose tissue, heart, skeletal muscle, lungs and myeloid cells that regulate metabolic pathways and energy homeostasis [1]. Eighteen KLFs have been identified thus far, all possessing a conserved C2H2‐type DNA‐binding zinc finger domain with a GC‐rich DNA‐binding sequence [2, 3]. Moreover, numerous studies have identified KLF variants associated with metabolic syndromes like type 2 diabetes (T2DM), maturity‐onset diabetes in the young (MODY) and increased body mass index (BMI) [4, 5]. For example, the variants KLF14, KLF11, KLF7 and KLF15 have been shown to be associated with T2DM, whereas KLF6, KLF7, KLF9, KLF13 and KLF15 were associated with increased BMI [1].

Many studies have demonstrated the role of KLF15 in the pathophysiology of multiple diseases, including metabolic syndromes [1]. KLF15 is ubiquitously expressed but is predominantly found in the kidneys and liver [6, 7]. The *KLF15* gene is located on chromosome 3q21‐q22 and is 416 amino acids long, comprising a disordered region (1–83 aa), a 9aa (transactivation domain) TAD motif (118–126 aa) [8], and three conserved C2H2‐type zinc finger (ZnF) domains (321–404) on its C‐terminal region [3, 6]. ZnF domains are linked linearly to bind GC‐rich nucleic acid sequences of varying lengths of target gene promoters, consequently regulating transcription [9]. Studies on hepatic cells demonstrated that KLF15 expression increases during the fasting state and is involved in regulating gluconeogenesis [7]. Additionally, it was the first KLF to be associated with metabolic dysfunction due to its role in regulating glucose transporter type 4 (GLUT4). GLUT4 is mainly expressed in muscle and adipose tissue and is the primary insulin‐responsive glucose transporter [10]. As a result, overexpression of KLF15 drives the transcription of GLUT4, significantly increasing cellular glucose uptake [11]. Moreover, during adipocyte differentiation, KLF15 inhibition negatively regulates peroxisome proliferator‐activated receptor γ (PPARγ) expression in 3 T3‐L1 preadipocytes, preventing adipogenesis [12]. Despite these findings, the KLF15–PPARγ promoter binding sites have not been explored [13]. In the liver, KLF15 tightly controls the homeostasis of both lipogenesis and gluconeogenesis. It does so by binding to the lipogenesis regulator liver X receptor/retinoid X receptor (LXR/RXR), which controls the lipogenic program that regulates the transcription levels of sterol regulatory element binding transcription factor 1 (Srebf1), subsequently influencing triglyceride levels in circulation [7, 14]. During fasting, LXR/RXR and KLF15 form a complex with receptor‐interacting protein 140 (RIP140), which in turn reduces the expression levels of sterol regulatory element binding protein 1c (Srebp‐1c). This consequently promotes the expression of lipogenic enzymes that mediate the switch from lipogenesis to gluconeogenesis [7, 15]. Studies on KLF15−/− hepatocytes demonstrated that alanine aminotransferase (ALT) activity, which is responsible for converting alanine to pyruvate, is reduced by ~50%, affecting the gluconeogenic pathway. Compared to control, KLF15‐deficient mice experienced severe hypoglycemia during overnight fasts [7, 15]. However, in the postprandial state, KLF15 levels are reduced, RIP140 is replaced with the coactivator steroid receptor coactivator‐1 (SRC‐1), and the Srebp‐1c promoter is activated [7].

Metformin is one of the primary drugs administered for glycemic control for individuals with T2DM [16]. Several studies have demonstrated the effect of metformin on KLF15 activity either directly or indirectly. Administering metformin to cultured hepatocytes reduced KLF15 expression due to the degradation and downregulation of KLF15 mRNA, reducing the activity of genes involved in gluconeogenesis and resulting in less glucose production by the liver [17]. In another study, metformin attenuated the risk of left ventricular hypertrophy due to the AA allele of KLF15, an inducer of branched‐chain amino acid (BCAA) catabolism [2]. Therefore, metformin may influence the activity of KLF15, affecting BCAA metabolism and potentially reducing the risk of cardiac hypertrophy [18]. The aforementioned studies highlight the critical role KLF15 plays in regulating genes involved in glucogenesis and maintaining blood sugar levels in T2DM. This further implicates the importance of KLF15 as a therapeutic target for drug development against the KLF15‐regulated pathway involved in metabolic dysfunction, including T2DM.

Despite the extensive studies on the functional role of KLF15 as a transcription factor, the binding mechanism between its ZnF domain and nucleic acids remains elusive. In this study, we illustrate the interaction dynamics between KLF15 ZnF domain bound to DNA and provide insight into the significant residues forming the KLF15 ZnF–DNA complex. These interactions were explored at the atomic level by modelling the KLF15 ZnF–DNA complex with AlphaFold 3.0 [19]. Additionally, alanine mutagenesis was utilised to identify crucial residues involved in the KLF15 ZnF–DNA complex. The conformational stability and dynamic features of the KLF15 ZnF–DNA complex were subjected to 100 ns molecular dynamic (MD) simulations. Moreover, we investigated the binding free energy using MM/GBSA to calculate binding energies, confirm critical KLF15 ZnF domain residues, and highlight therapeutic hotspots. In summary, our research offers structural insights into the KLF15 ZnF–DNA interaction, which could potentially be instrumental in developing compounds for treating metabolic syndrome, including T2DM.

## 2 Materials and Methods

### 2.1 Structural Validation of AlphaFold 3.0 Models

Since the three‐helix KLF15 ZnF domain structure in apo state or in a complex with DNA has not been solved experimentally by nuclear magnetic resonance (NMR) or X‐ray crystallography AlphaFold 3.0 [19] was used to model the KLF15 ZnF bound to a DNA complex. Initially, to validate the accuracy of AlphaFold 3.0 in modelling a protein‐DNA complex, we retrieved the KLF4 ZnF domain amino acid sequence and DNA sequence used to solve the X‐ray crystal structure of the KLF4 ZnF‐DNA structure (PDB ID: 2WBU) [20] and used AlphaFold 3.0 to model the same KLF4‐DNA complex. In addition, to further validate the accuracy of AlphaFold 3.0, the modelled KLF15 ZnF domain was modelled against the solved NMR structure of KLF15 ZnF domain α − helix (H2) structure from residues 347 to 380 (PDB ID: 2ENT) [21].

### 2.2 Structural Modelling of KlF15‐Zinc Finger Domain DNA Complex

The 416‐amino‐acid sequence of KLF15 was retrieved from UniProt (accession number Q9UIH9). The KLF15 ZnF domain (residues 321–403) was subjected to highly accurate structure modelling with AlphaFold 3.0 as it can predict the joint structure of complexes such as nucleic acid (RNA and DNA), proteins and small molecules [22]. Since KLF15‐ZnF binds to GC base pairs [23, 24], the KLF15‐ZnF domain was modelled with the 5′GAGGCGTGGC3′ DNA sequence from the solved KLF‐DNA X‐ray crystal structure (PDB ID: 2WBU) [20, 25]. The predicted per‐residue scores on the lDDT‐Cα metric (pLDDT) scores were used as a model validation criterion. The final structure was minimised using the steepest descent and conjugate gradient algorithms for 1000 and 300 steps using Chimera software [26]. The interaction analysis between KLF15 ZnF domain residues and DNA was conducted with the structural analysis server PDBsum (https://www.ebi.ac.uk/thornton‐srv/databases/pdbsum/) [27].

### 2.3 KLF15 ZnF Domain–DNA Docking and *in Silico* Alanine Substitution

The impact of missense mutations on protein–DNA interactions is estimated and interpreted using PremPDI. PremPDI exploits integrated molecular mechanics force fields and fast side‐chain optimization algorithms to accurately estimate the impact of any substitution on the binding of DNA and protein. In this study, PremPDI (https://lilab.jysw.suda.edu.cn/research/PremPDI/) was used to compute the impact of each alanine substitution on the binding of KLF15 and DNA [28]. Alanine was substituted at each position to determine essential DNA recognition and binding residues. The highest impacted residues were shortlisted and subjected to structural analysis and MD simulations.

### 2.4 Dynamics of the KLF15 ZnF Domain–DNA Complex

To assess the deleterious substitutions of the KLF15 ZnF domain residues and their impact on DNA binding. The structural dynamics of the KLF15 ZnF domain‐DNA were explored with AMBER20 using FF19SB [29, 30]. Each complex was solvated with an optimal point charge (OPC) water model of 10 Å, and six sodium ions were added for the neutralisation of each complex, followed by gentle minimisation of 6000 and 3000 steps by employing steepest descent and conjugate gradient algorithms and heated up to 300 K. The pressure control was applied using the Berendsen barostat, temperature control was maintained with the Langevin thermostat, and heat control was managed through periodic velocity rescaling. These methods ensure stable simulation conditions and accurate representation of the thermodynamic properties of the system. The Particle Mesh Ewald (PME) algorithm was employed to compute the long‐range electrostatic interactions. For van der Waals interactions and short‐range Coulombic interactions, a cutoff value of 1.4 nm was set. After 50 ns of equilibration, a 100‐ns production run was completed, which was analysed via the CPPTRAJ and PTRAJ integrated modules of AMBER [31]. Structural stability was calculated through root mean square deviation (RMSD) estimation, while the residue flexibility was calculated as a root mean square fluctuation (RMSF) function. Other post‐simulation analyses of the trajectories, including the gyration (Rg) radius, were also performed using the simulation trajectories.

### 2.5 Assessment of the Binding Free Energy of the KLF15 ZnF Domain–DNA Complex

The binding conformation and energy can be precisely estimated through MM/GBSA calculation. For this purpose, each complex, that is, WT and alanine‐substituted residues, was subjected to free energy calculation using the MMPBSA.py script [32]. For this purpose, the last 5000 frames from each trajectory, which make up 100 ns, were used for the free energy calculation. Mathematically, the following equation was used to estimate the binding energy:

$$\begin{array}{c} ∆G_{\mathrm{net}\;\text{binding energy}}=∆G_{\text{complex binding energy}}\\ \;-\left[∆G_{\text{receptor binding energy}}+∆G_{\text{ligand binding energy}}\right] \end{array}$$

Each of the above components of net binding energy can be split as follows:

$$G=G_{\text{bonded}}+G_{\mathrm{van}\;\mathrm{der}\;\text{Waals}}+G_{\text{polar solvation energy}}+G_{\text{non}-\text{polar solvation energy}}$$

## 3 Results and Discussion

### 3.1 Structural Validation KL15 and KLF15‐DNA Interactions Modelling

The KLF15 ZnF domain comprises three alpha helices interacting with DNA; however, the entire zinc finger domain structure has not been determined experimentally. Therefore, we utilised the AlphaFold 3.0 [19] structural prediction tool to determine the KLF15 C2H2‐type ZnF domain three‐dimensional structure in a complex with DNA. AlphaFold 3.0 is a deep‐learning framework that predicts the structures of biomolecular complexes, including proteins, DNA and RNA. In addition, AlphaFold 3.0 can model biomolecular interactions, such as protein–protein, protein‐DNA, protein‐RNA and protein‐ligand interactions.

**FIGURE 1:**
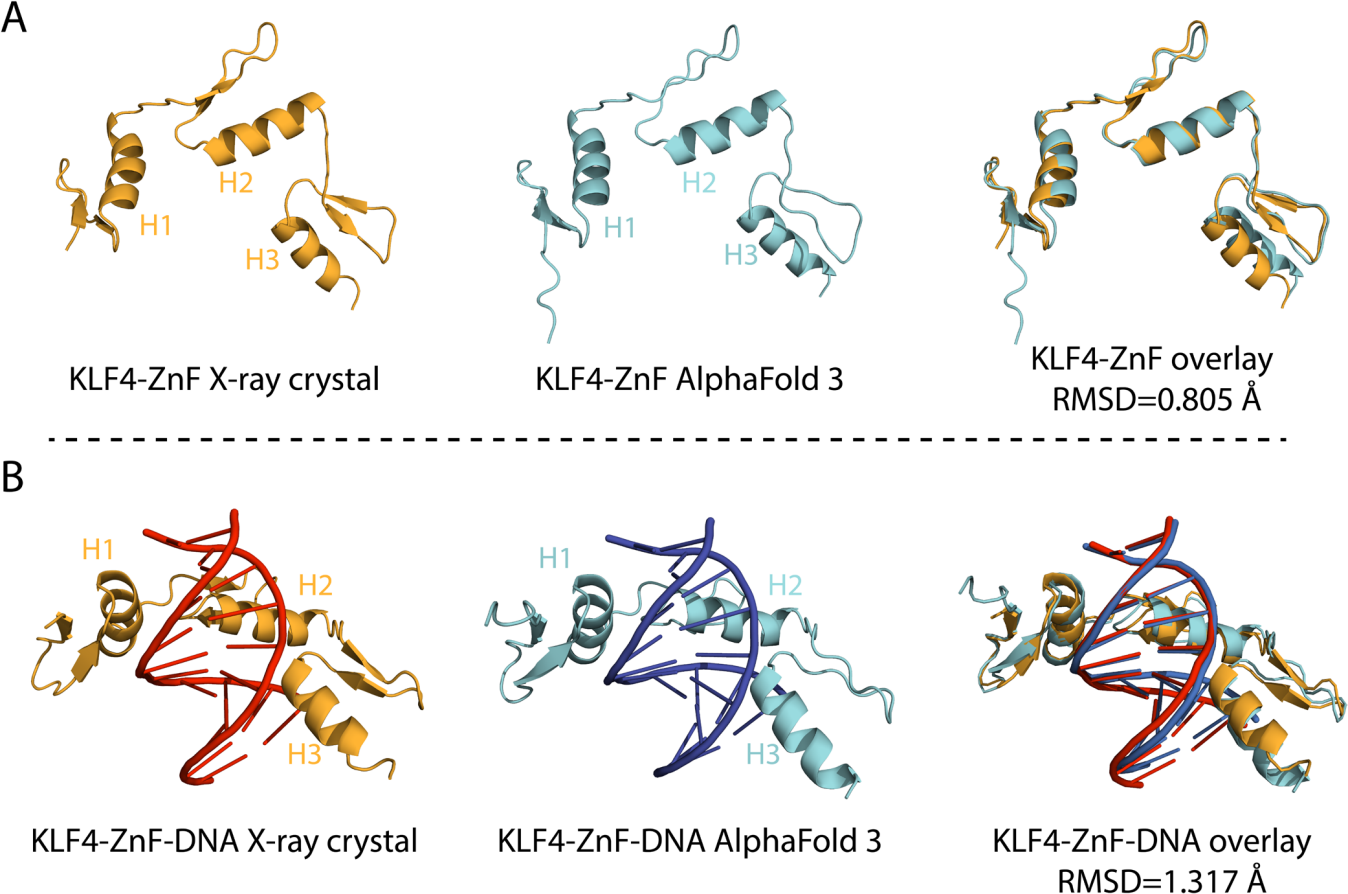
Structural validation of KLF4 ZnF X‐ray and AlphaFold 3.0 modelled KLF4 ZnF structures. (A) X‐ray crystal structure KLF4 ZnF domain (orange) (PDB ID: 2WBU) overlayed with KLF4 ZnF AlphaFold 3.0 modelled (cyan) structure in apo states. (B) The KLF4 ZnF domain DNA complex superimposed with the AlphaFold 3.0, which modelled the KLF4 ZnF domain in a complex with DNA.

Thus, to validate the accuracy of AlphaFold 3.0 structural modelling of the KLF15 and the KLF15‐ZnF DNA binding, the experimentally determined X‐ray crystal structure of the KLF4‐ZnF domain in complex with DNA (PDB ID: 2WBU) [20] was compared with the KLF4‐ZnF DNA complex modelled with AlphaFold 3.0 (Figure 1A,B). The X‐ray crystal KLF4 ZnF domain in its apo state and the KLF4 ZnF domain AlphaFold 3.0 modelled structure present three α‐helices characteristic of the C2H2 zinc finger fold (Figure 1A). The overlay of the KLF4 ZnF domain X‐ray crystal and the modelled AlphaFold 3.0 structures presented a high degree of structural similarity, with an RMSD of 0.805 Å. Furthermore, the X‐ray crystal structure of the KLF4 ZnF domain bound to DNA (PDB ID: 2WBU) overlayed with AlphaFold 3.0 modelled KLF4 ZnF–DNA structure resulted in an RMSD of 1.317 Å. AlphaFold 3.0 accurately predicts the protein fold and protein‐DNA interactions, indicating a strong agreement between the experimental and predicted models.

**FIGURE 2:**
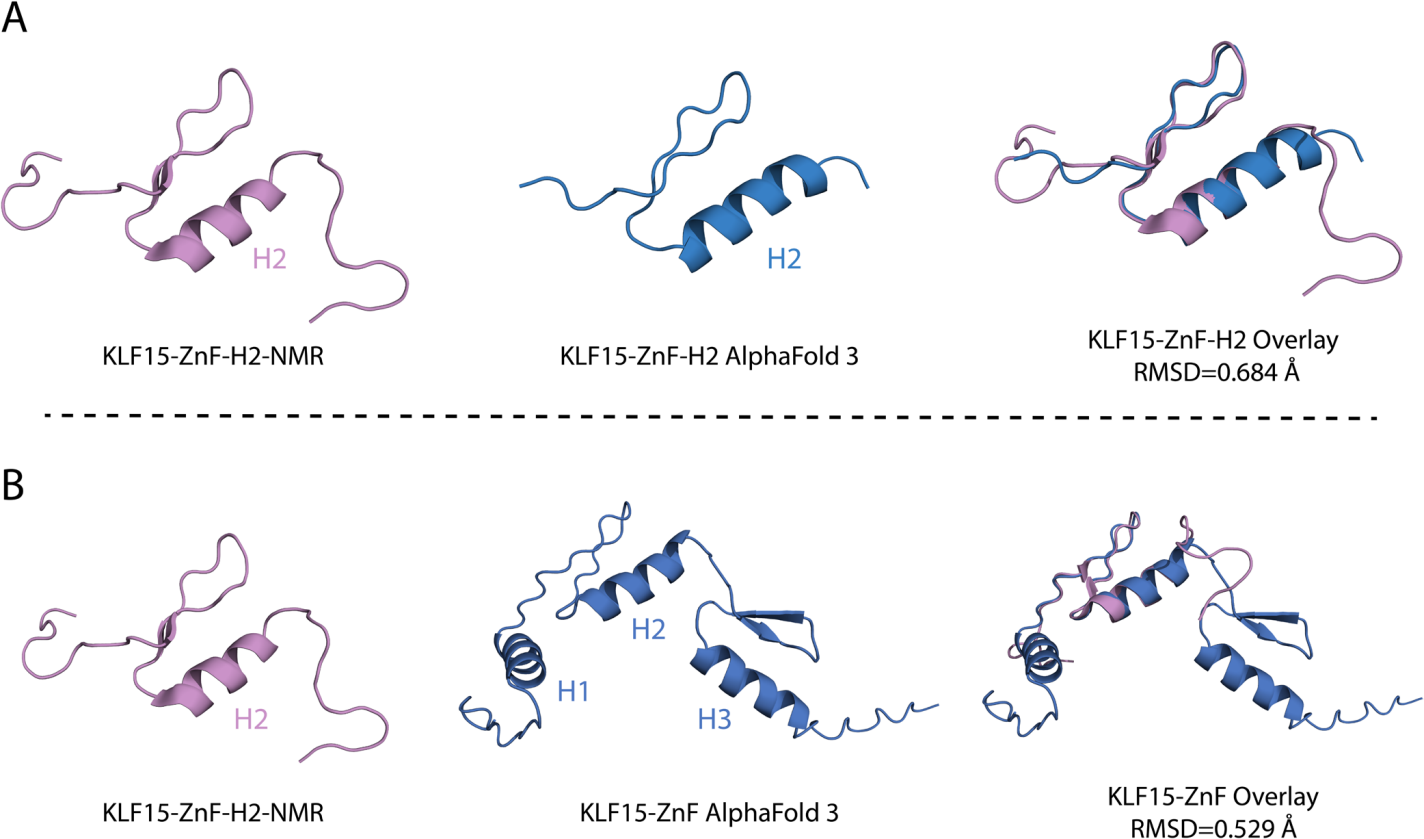
Structural validation of KLF15 ZnF NMR and AlphaFold 3.0 modelled KLF15 ZnF structures. (A) NMR KLF15 ZnF H2 domain (purple) (PDB ID: 2ENT) superimposed with KLF15 ZnF H2 AlphaFold 3.0 modelled (blue). (B) NMR KLF15 ZnF H2 domain superimposed with the full KLF15 ZnF AlphaFold 3.0 modeled.

The three‐helix ZnF domain of KL15 structure in the apo state or bound to DNA has not been solved experimentally. However, the second helix (H2) structure of the KLF15 ZNF domain from residues 347 to 380 has been determined by NMR (PDB ID: 2ENT). As such, the KLF15 ZnF domain H2 NMR structure was used to validate the structure of the KLF15 ZnF domain in the apo state modelled with AlphaFold 3.0 (Figure 2A,B). Initially, the AlphaFold 3.0 modelled structure of the H2 KLF15 ZnF domain aligned with the H2 NMR structure demonstrated high structural similarity with an RMSD of 0.684 Å. Furthermore, AlphaFold 3.0 modelled the structure of all three alpha‐helices (H1, H2 and H3) in the KLF15‐ZnF domain aligned with the KLF15 ZnF domain H2 NMR structure (Figure 2B), indicating good agreement between the experimental and modelled structures (RMSD 0.529 Å).

### 3.2 KLF15 Zinc Finger Domain DNA Binding With AlphaFold 3.0

**FIGURE 3:**
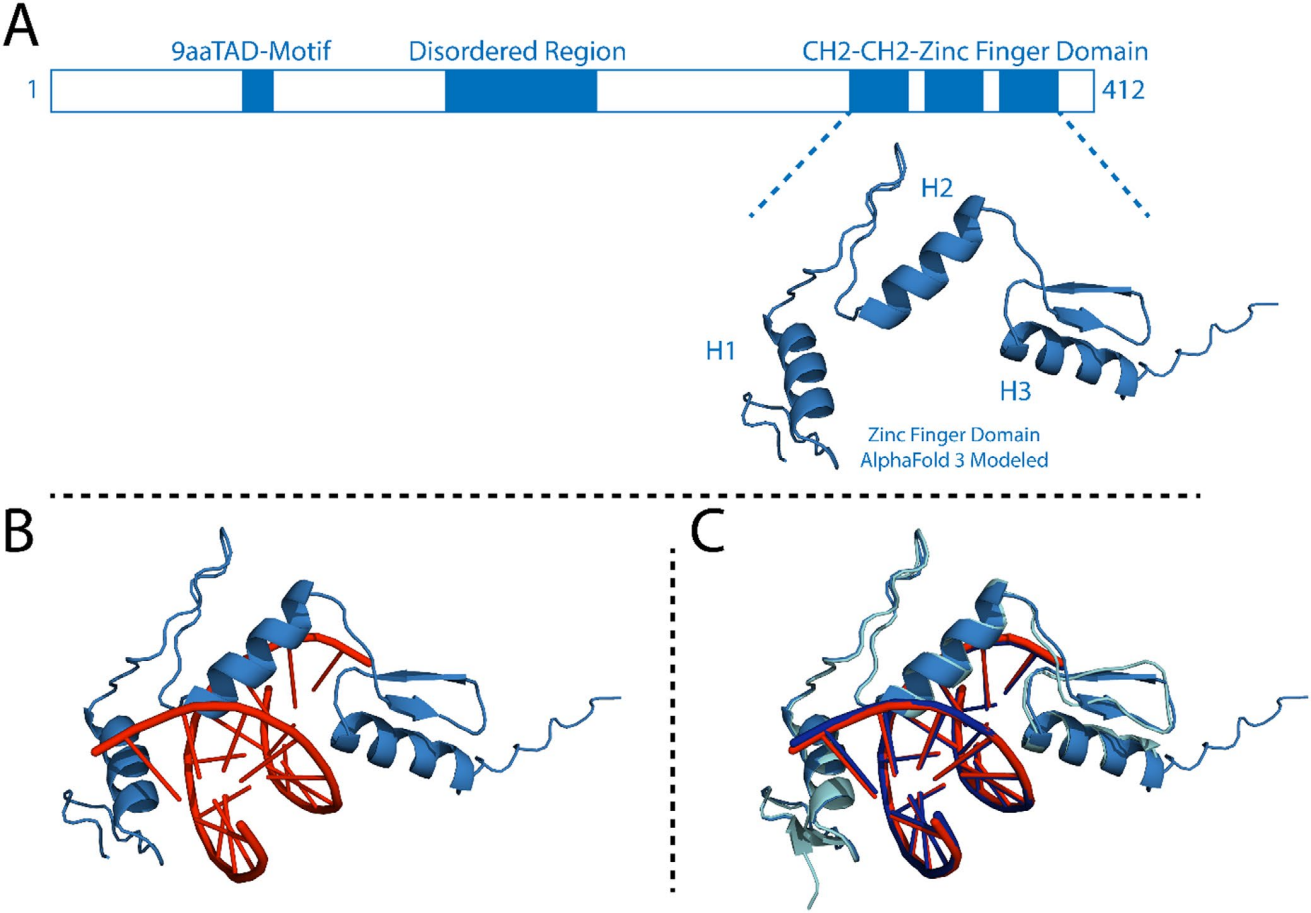
Structural organisation and 3D structure of KLF15. (A) represents the structural organisation of KLF15, (B) shows the Alphafold 3.0 modelled 3D structure of KLF15 ZnF‐DNA complex. (C) Superimposed Alphafold 3.0 modelled KLF15 ZnF‐DNA complex (blue) with KLF4 ZnF‐DNA complex AlphaFold 3.0 modelled (cyan).

KLF15 is 416 amino acids in length, comprising a disordered region (1–83 aa), a 9aaTAD motif between residues 118 and 126, and three C2H2‐type zinc finger domains corresponding to positions 321–404 aa (Figure 3A). Since no experimental data have demonstrated the interaction between the KLF15 ZnF domain and DNA, we employed the AlphaFold 3.0 protein‐DNA interactions modelling to build the KLF15 ZnF domain DNA complex. KLF15 ZnF domains predominantly interact with GC‐rich DNA sequences. Thus, the KLF15 ZnF domain sequence from residue 321 to 404 was modelled with a GC‐rich DNA sequence extracted from the X‐ray crystal KLF4‐ZnF DNA complex DNA (PDB ID: 2WBU) [20] (Figure 2B). The overlay of the modelled KLF15 ZnF–DNA complex and the X‐ray crystal structure of the KLF4 ZnF–DNA exhibited an RMSD of 4.4 Å (Figure 2C), indicating the modelled KLF15 ZnF domain binding to the GC‐rich DNA represents a structural similarity with the experimentally solved KLF4 ZnF–DNA structure (PDB ID: 2WBU).

**FIGURE 4:**
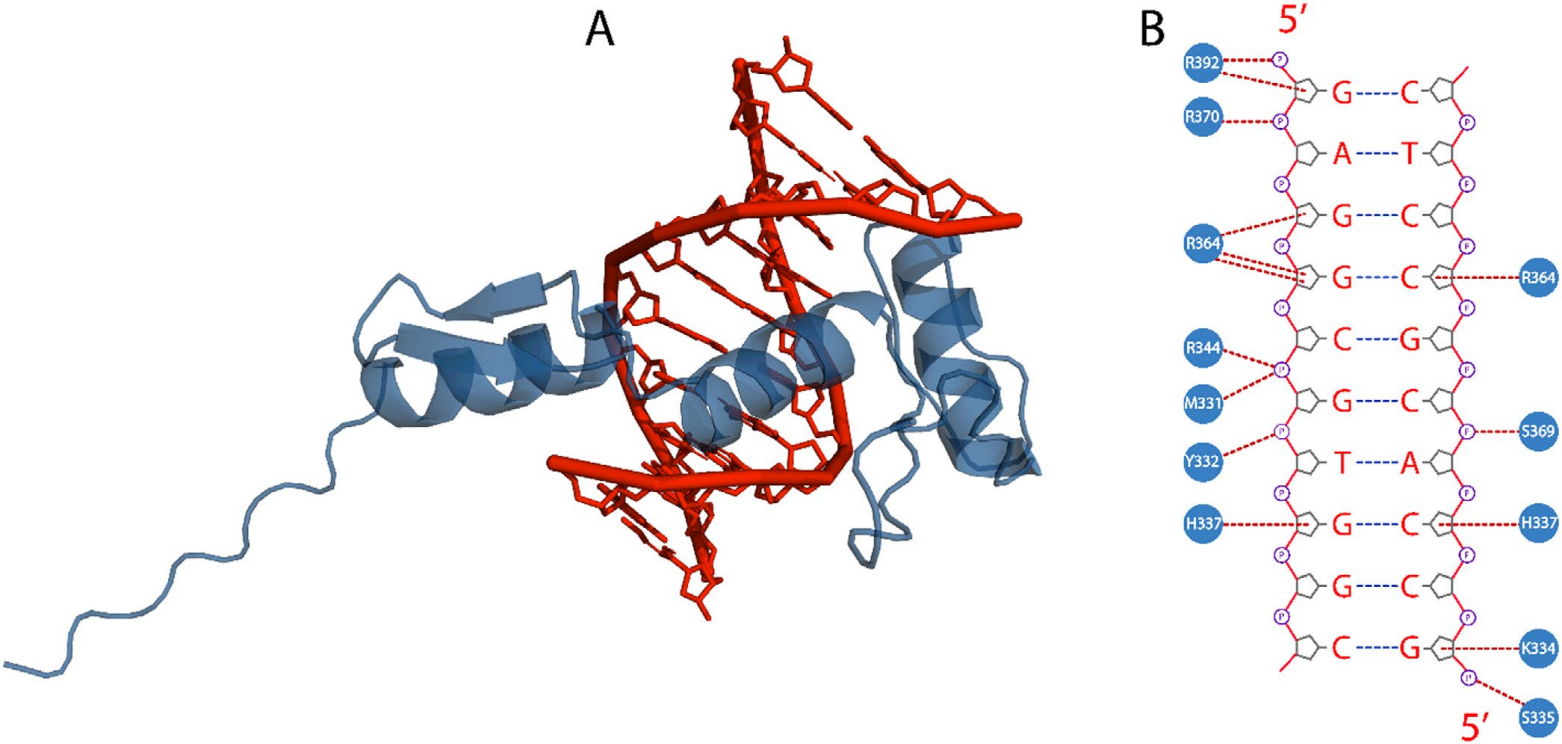
Binding mode of KLF15 ZnF domain and DNA. (A) demonstrates the binding interface between KLF15 and DNA, while (B) depicts the interaction pattern of KLF15 ZnF domain and DNA.

The KLF15 ZnF domain–DNA complex demonstrated key residues forming H‐bonds with the nucleotides. The interactions included H‐bonds formed by K330, M331, Y332, K334, S335, R344, R364, R370, S369 and R392 (Figure 4B). Predominantly, the residues forming H‐bonds with DNA were positively charged amino acids, which are similar to the KLF4 ZnF–DNA complex, where residues H446, R473, R479 and R501 formed H‐bonds with the nucleotide base pairs [20]. Furthermore, PDBsum [27] structural analysis demonstrated that K330, H341, R344 and R370 formed charge–charger interactions (salt bridges) with the DNA phosphate backbone, possibly to stabilise further the KLF15 ZnF–DNA complex [33]. Since H‐bonds play a critical role in the specificity and stability of the KLF15 ZnF–DNA complex, they also contribute to the specificity of the interactions by recognising DNA sequences. Therefore, changing any amino acid residue involved in the KLF15 ZnF–DNA complex would provide insight into the importance of the binding mechanism.

### 3.3 *In Silico* Alanine Mutagenesis of the KLF15 Zinc Finger Domain

In silico, alanine mutagenesis is an approach to interpret essential residues in protein–protein and protein–DNA molecular interactions. Furthermore, such residues may be essential in protein or DNA binding recognition and inhibition [34]. The PremPDI [28] algorithm uses molecular mechanics force fields and fast side‐chain optimization with parameters optimised on experimental sets to estimate binding affinity change and determine the deleterious effects of alanine substitutions. Twelve residues of the KL15 ZnF domain formed H‐bonds with the DNA molecule (Figure 4). The KLF15 ZnF–DNA complex residues were subjected to alanine mutagenesis, where the binding affinities (ΔΔG) changes were calculated upon alanine substitution. The ΔΔG involves comparing the predicted binding free energies of the KLF15 ZnF WT residues with alanine substitution to measure if the substitution is deleterious (Table 1).

**TABLE 1:** Binding affinity change and deleterious effects of alanine substitutions defined by ΔΔG (kcal/mol) at different positions predicted by PremPDI. The residues highlighted in red represent the most deleterious alanine substitutions.

Mutation	ΔΔG (kcal/mol)	Interface	Deleterious
K334A	1.70	Yes	Yes
R344A	1.63	Yes	Yes
Y332A	1.61	Yes	Yes
R392A	1.61	Yes	Yes
H337A	1.46	Yes	Yes
R364A	1.23	Yes	Yes
R372A	1.20	No	Yes
R370A	0.97	No	No
M331A	0.87	Yes	No
S369A	0.86	Yes	No
R373A	0.81	No	No
S335A	0.34	No	No

K334A induced the most significant fold change in the binding affinity ΔΔG = 1.70 kcal/mol, with deleterious functional effects on binding the KLF15 ZnF domain to DNA. K334 is in the solvent‐exposed region of the H1 ZnF domain helix, forming an H‐bond between the K334 side chain and DNA. Therefore, losing the H‐bond may have led to a loss in the overall stability of the KLF15 ZnF domain–DNA complex. Residue R344, also situated in the solvent‐exposed region of the helix, exhibited a deleterious effect when substituted with alanine, with a ΔΔG = 1.63 kcal/mol. Furthermore, the alanine substitution of surface residues Y332 and R392 resulted in a deleterious effect on the KLF15–DNA complex, causing a loss of stability (ΔΔG = 1.61 kcal/mol). The remaining two residues with weaker deleterious effects when substituted with alanine included H337 (ΔΔG = 1.46 kcal/mol) and R364 (ΔΔG = 1.23 kcal/mol). H337 and R364 are surface residues on the zinc finger domain H1 and H2, respectively. However, their position in the centre of the helix may have compensatory effects from other non‐covalent interactions that can reduce the deleterious effect of alanine substitution. Studies have demonstrated that mutations in the ZnF domains of KLF proteins significantly impact their DNA‐binding capabilities and regulatory functions. In KLF4, the E476D and R501A mutations in the ZnF H2 and ZnF H3, respectively, reduce liquid–liquid phase separation (LLPS) with DNA, crucial for chromatin organisation, thereby impairing DNA‐binding domain (DBD): DNA condensation [25]. In KLF1, the E339D and E325K mutations within the ZnF domain lead to severe anaemia by altering DNA‐binding specificity, activating ectopic sites and inducing neomorphic gene expression [35].

On the other hand, residues R370, M331, S369, R373 and S335 did not present deleterious effects when substituted with alanine (Table 1). Although alanine substitutions would result in the loss of H‐bonds between the KLF15 ZnF residues and DNA, in some cases, other interactions such as electrostatic forces, van der Waals interactions and hydrophobic effects can compensate for the loss of H‐bonds [36]. Although the PremPDI server predicted the energy changes upon the alanine substitution, the analysis is based on a single KLF15 ZnF domain DNA complex structure. Therefore, to determine more accurately the impact of the top four most deleterious residue substitutions, K334A, R334A, Y332A and R392A (Table 1), we used a MD simulation, a more reliable tool, to elucidate the structural stability of the KLF ZnF domain interacting with DNA.

### 3.4 Dynamic Analysis of the KLF15 ZnF–DNA Complex

The conformational dynamics of the KLF15 ZnF–DNA complexes (WT and K334A, R334A, Y332A and R392A) were assessed by running 100‐ns MD simulations. The RMSD trajectories of the Cα‐atoms represent the degree to which the protein conformation has changed over time. Meanwhile, the Rg trajectories reveal the degree of compactness of the KLF15 ZnF–DNA complexes. In addition, the RMSF values of the Cα‐atoms reflect the residual flexibility of KLF15 ZnWT and were compared with K334A, R334A, Y332A and R392A substitutions. To corroborate the impact of each substitution on the KLF15 ZnF structure, we applied the MM/GBSA method to calculate the total binding free energy (ΔG) to DNA.

**FIGURE 5:**
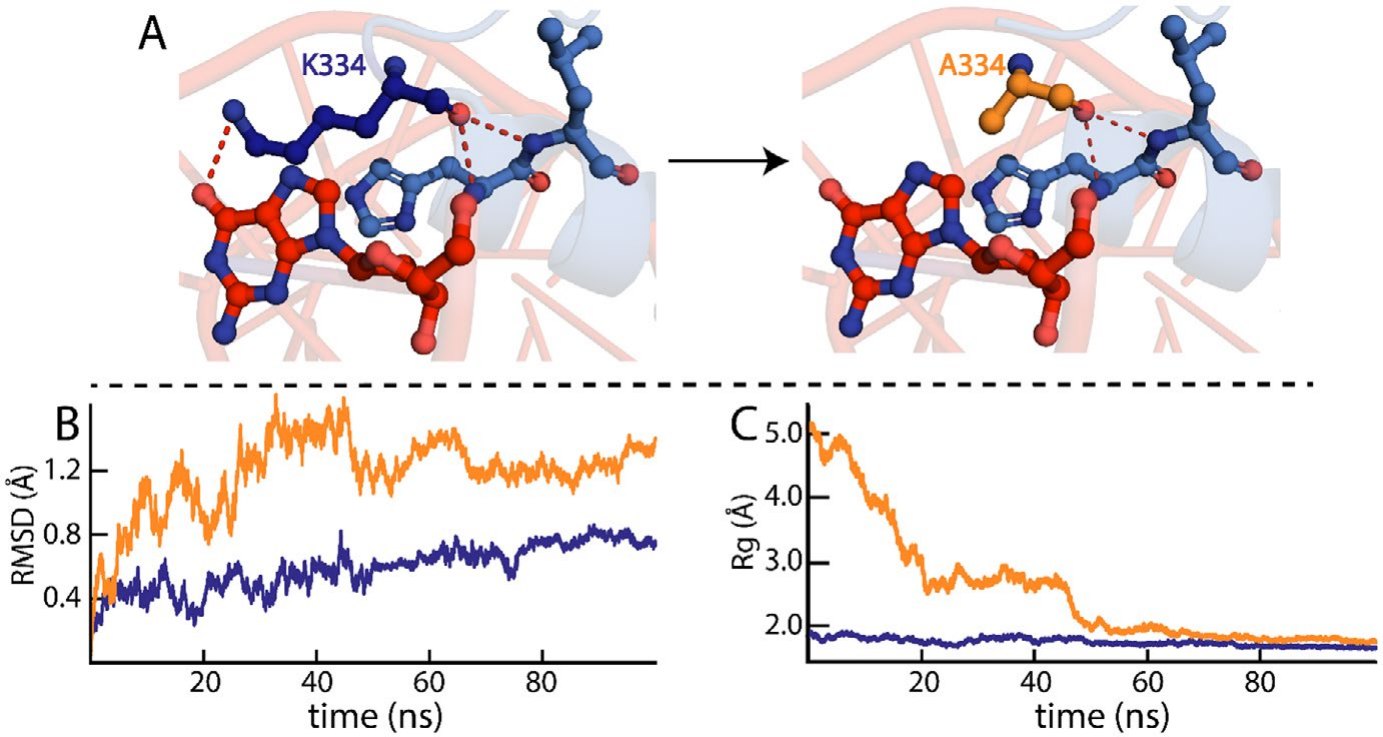
Dynamic stability of the K334A KlF15–DNA complex. (A) demonstrates the loss of the H‐bond when K334 is substituted with alanine. (B) RMSD trajectories of WT (blue) and K334A (orange) KLF15 ZnF domains complexed with DNA. (C) depicts the Rg compactness of WT (blue) and K334A (orange) KLF15 ZnF domains complexed with DNA.

The RMSD trajectories of the WT KLF ZnF domain in complex with DNA were analysed to evaluate its dynamic behaviour throughout the 100‐ns MD simulation (Figure 5B). The RMSD of the WT KLF ZnF–DNA complex begins at approximately 0.3 Å at 0 ns and displayed an initial increase from 0.6 to 0.7 Å in the initial 10 ns of the simulation. Then, the RMSD fluctuated from 10 to 20 ns, maintaining a value around 0.6–0.7 Å, then decreasing to 0.3 Å, indicating the initial equilibration phase of the WT KLF ZnF–DNA complex. From 20 ns to 42 ns, the RMSD increased from 0.3 to 0.8 Å, with fluctuations at 25 ns, after which the RMSD decreased to 0.4 Å at 45 ns. Subsequently, from 45 to 90 ns, the WT KLF ZnF–DNA complex RMSD increased from 0.4 to 0.8 Å, with the remainder of the 100 ns simulation, the RMSD stabilised at 0.7 Å. The WT KLF ZnF–DNA complex Rg plot (Figure 5C) demonstrated a slight fluctuation between 1.7–2.0 Å, demonstrating a compact and stable conformation during the 100‐ns simulation. The Rg data demonstrated a stable and compact state, with minimal deviations observed throughout the 100 ns simulation, indicating the WT KLF15 ZnF–DNA complex is in a stable compact and stable conformation.

The RMSD of the K334A KLF15 ZnF domain bound to DNA exhibited a highly dynamic complex throughout the 100‐ns simulation (Figure 5B). The RMSD trajectory displayed a significant perturbation in the initial 35 ns of the simulation, with an increase in RMSD from 0.4 to 1.6 Å, with fluctuations in RMSD at 17 and 22 ns. The fluctuations can result from the K334A KLF15 ZnF‐DNA complex conformation deviating significantly in the initial 35 ns of the simulation. The initial deviation demonstrates that the K334A KLF15 ZnF‐DNA complex is equilibrating and exploring conformational space. From 35 to 45 ns, the K334A KLF15 ZnF‐DNA complex plateaued at 1.6 Å, following a decrease to 1.2 Å at 50 ns. Subsequently, from 50 ns to the remainder of the 100 ns simulation, the K334A KLF ZnF‐DNA complex averaged an RMSD between 1.2 and 1.4 Å. Overall, the K334A KLF ZnF–DNA complex presented an average RMSD of between 1.0 and 1.4 Å, with significant structural fluctuations over the simulation compared to the RMSD of the WT KLF ZnF–DNA complex (0.8 Å).

Furthermore, the K334A KLF15 ZnF–DNA complex Rg displayed a variable pattern to the WT KLF15 ZnF–DNA complex (Figure 5C). In the initial phase of the 100 ns simulation, the RG of the K334A KLF ZnF–DNA complex demonstrated a decrease from 5 to 1.9 K334A KLF ZnF–DNA complex in the first 20 ns, after which, from 20 to 50 ns, the K334A KLF15 ZnF–DNA complex the Rg remains at 2.9 Å. The high Rg fluctuation experienced by the K334A KLF15 ZnF–DNA complex in the initial phase of the simulation indicated that a loss of compactness could result from conformational sampling. Subsequently, from 50 ns till the completion of the simulation, the Rg decreased to 1.8 Å, similar to the Rg of the WT KLF15 ZnF–DNA complex. The fluctuations in the RMSD and Rg observed corroborate the deleterious effect measured by K334 alanine substitution (ΔΔG = 1.63 kcal/mol). In addition, the loss of the H‐bond between K334 and the guanine nucleotide (Figure 5A) might have affected the KLF15 ZnF–DNA complex, resulting in the loss of compactness demonstrated by the Rg fluctuations.

**FIGURE 6:**
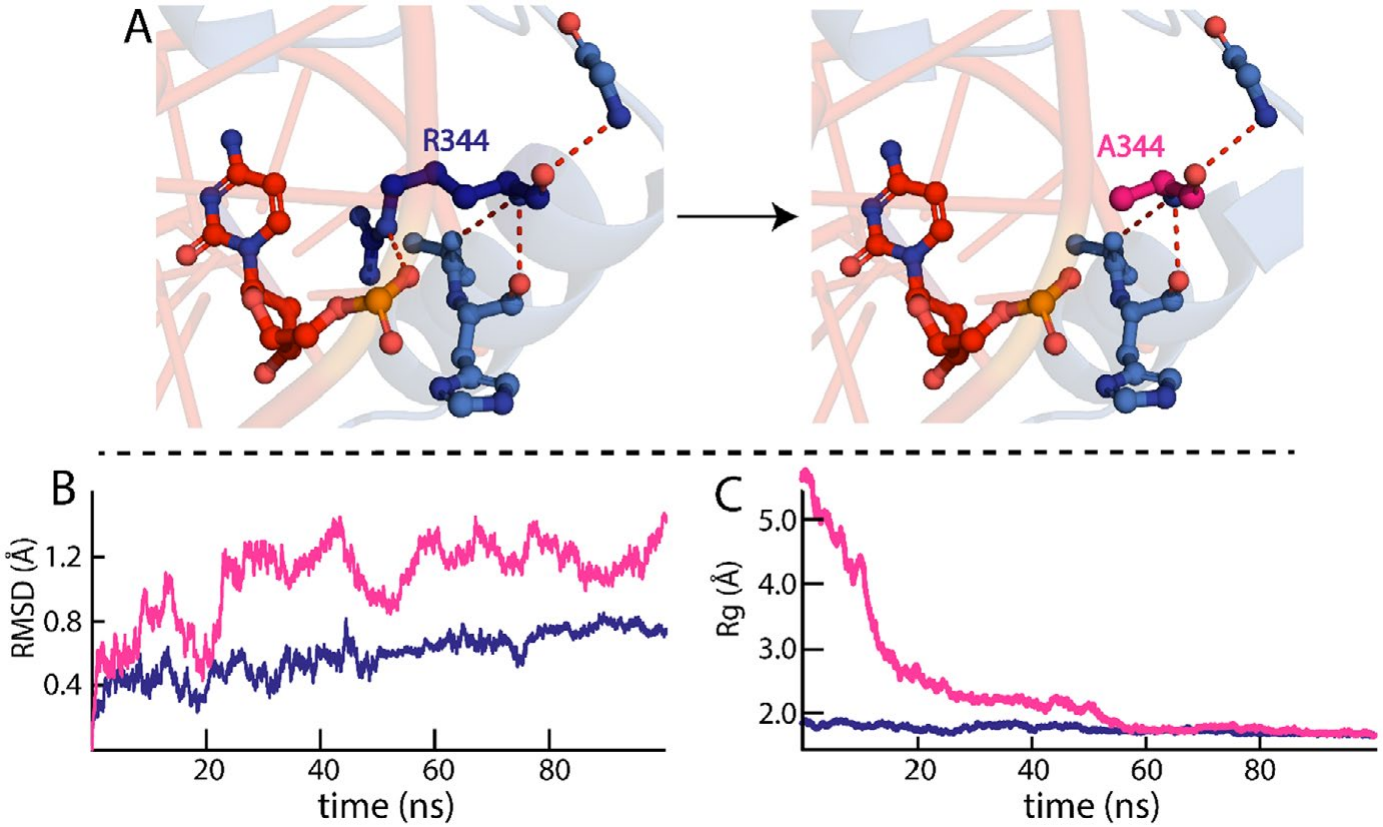
Dynamic stability of the R344A KlF15–DNA complex. (A) demonstrates the loss of the H‐bond when R344 is substituted with alanine. (B) RMSD trajectories of WT (blue) and R344A (magenta) KLF15 ZnF domains complexed with DNA. (C) Depicts the Rg compactness of WT (blue) and R344A (magenta) KLF15 ZnF domains complexed with DNA.

Compared to the WT KLF15 ZnF domain–DNA complex, the R344A KLF15 ZnF domain–DNA complex exhibited high RMSD fluctuations during the 100‐ns simulation, signifying structural instability (Figure 6B). During the initial 5 ns of the simulation, the RMSD increased from 0.4 to 0.6 Å, and then from 10 to 15 ns, the RMSD increased to 1.1 Å. Subsequently, from 15 to 22 ns, the R344A KLF15 ZnF domain–DNA fluctuates between 0.4 and 1.2 Å, indicating the system is in the equilibration phase. From 22 ns till the end of the simulation, the RMSD trajectories kept fluctuating between 1.0 and 1.3 Å, exhibiting a constant deviation in the RMSD indicative of the instability of the R344A KLF ZnF domain–DNA complex. Furthermore, the R344A KLF ZnF domain–DNA complex demonstrated a higher average RMSD (1.1 Å) than the WT complex (0.8 Å), whereby the instability demonstrated by the RMSD profile of the R344A KLF ZnF domain–DNA complex may have resulted from the loss of the H‐bond between R344 and the DNA molecule (Figure 6A).

Further substantiating the alanine substitution effect, the Rg analysis of the R344A KLF15 ZnF domain–DNA complex presented fluctuating trajectories during the 100‐ns simulation. In the initial phase of the simulation, the Rg trajectories start at 6.0 Å, dropping sharply to 2.5 Å at 20 ns, with KLF15 ZnF and DNA coming together and forming a stable complex, where the interaction may lead to the compactness of the complex. From 20 to 55 ns, the Rg trajectories decrease from 2.5 Å to 1.7 Å, which might indicate that the DNA is further compacting around the KLF15 ZnF domain. From 55 ns to the remainder of the 100 ns simulation, the Rg trajectories remained at an average of 1.7 Å, similar to the WT KLF15 ZnF domain–DNA complex Rg trajectories, demonstrating the complex has reached a stable and compact conformation. The loss of compactness in the initial phase of the simulation Rg trajectories and the instability shown in the RMSD profile support the deleterious effect of the alanine substitution (ΔΔG = 1.63). Additionally, the loss of the H‐bond indicates that the complex lost its compactness due to the alanine substitution.

**FIGURE 7:**
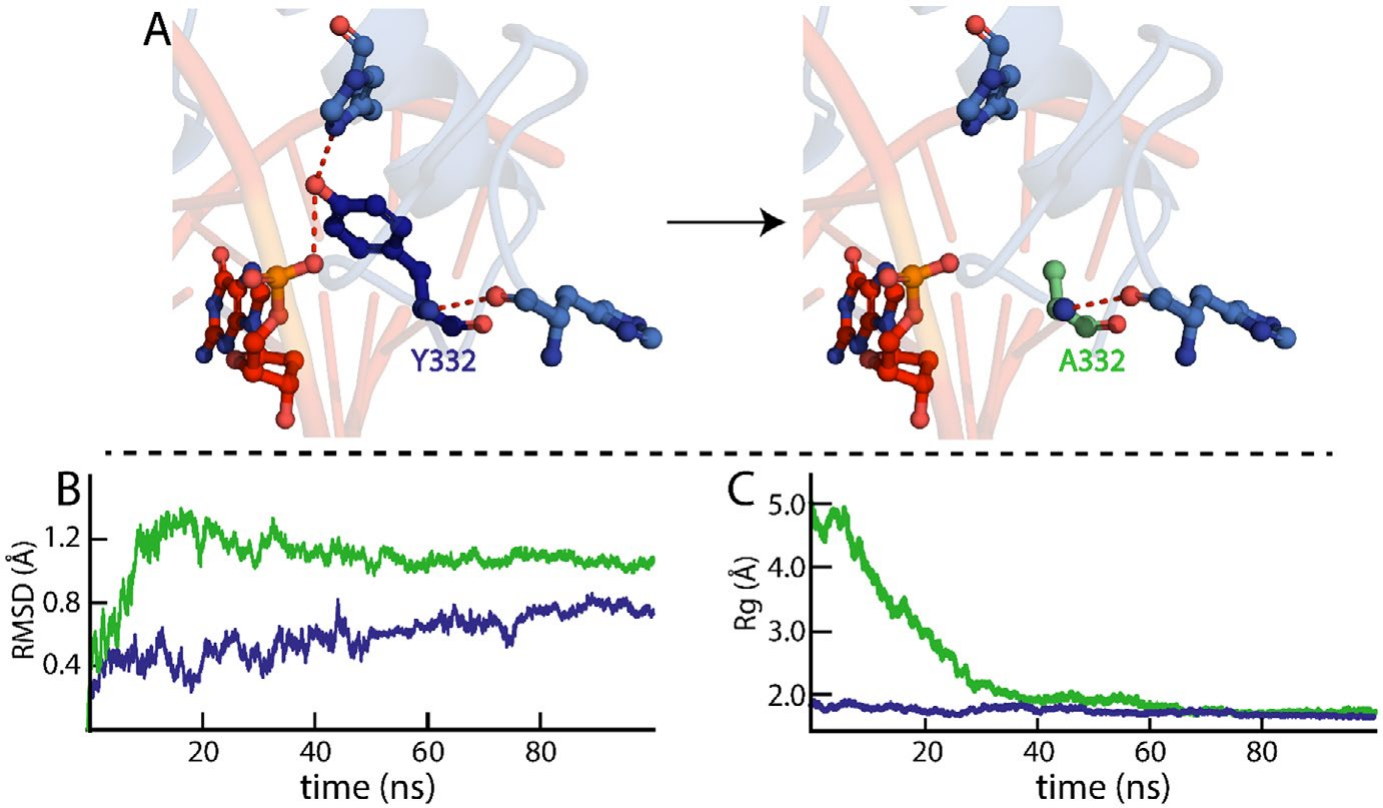
Dynamic stability of the Y332A KlF15–DNA complex. (A) demonstrates the loss of the H‐bond when Y332 is substituted with alanine. (B) RMSD trajectories of WT (blue) and Y332A (green) KLF15 ZnF domains complexed with DNA. (C) depicts the Rg compactness of WT (blue) and Y332A (green) KLF15 ZnF domains complexed with DNA.

The alanine substitution of Y332 resulted in the loss of two H‐bonds, one with K333 on H1 of the ZnF domain and one with the phosphate group of the T7 nucleotide (Figure 6A). This may have affected the stability of the Y332A KLF15 ZnF domain–DNA complex, as observed from the RMSD trajectories (Figure 7B). The RMSD trajectories (Figure 7B) showed an increase in RMSD of 1.3 Å within the first 20 ns of the simulation, demonstrating that the Y332A KLF15 ZnF domain–DNA complex is undergoing initial structural rearrangements during the equilibration phase. Subsequently, from 20 ns till the end of the simulation, the RMSD values fluctuated between 1.1 and 1.2 Å. Although the RMSD of the Y332A KLF15 ZnF domain–DNA complex remains constant through the later stages of the simulation, the higher RMSD of the Y332A KLF15 ZnF domain bound to DNA demonstrated a more flexible complex than the WT KLF15 ZnF domain bound to DNA (Figure 7B).

Furthermore, the Rg trajectories measured the structural compactness of the Y332A KLF15 ZnF domain–DNA complex (Figure 7B). The Rg trajectories of the Y332A KLF15 ZnF domain–DNA start higher than the WT KLF15 ZnF domain–DNA complex (5.0 Å), representing a less compact complex, which could be due to the variant disrupting initial interactions or causing structural instability. The Rg decreased significantly between 0 and 40 ns, from 5 to 2 Å, exhibiting the Y332A KLF15 ZnF domain–DNA complex in a more compact state. Subsequently, from 40 ns till the end of the simulation, the Rg trajectories remained on an average of 1.7 Å, similar to the WT KLF15ZnF domain–DNA complex. The loss of structural compactness can be attributed to a compensatory mechanism of interaction, whereby hydrophobic, van der Waals or charge–charge interactions compensated for the loss of the H‐bonds [37, 38]. In addition, the loss of stability of Y332 (ΔΔG = 1.61 kcal/mol) from the alanine substitution (Table 1) indicates the effect on the compactness of the Y332 KLF15 ZnF domain–DNA complex.

**FIGURE 8:**
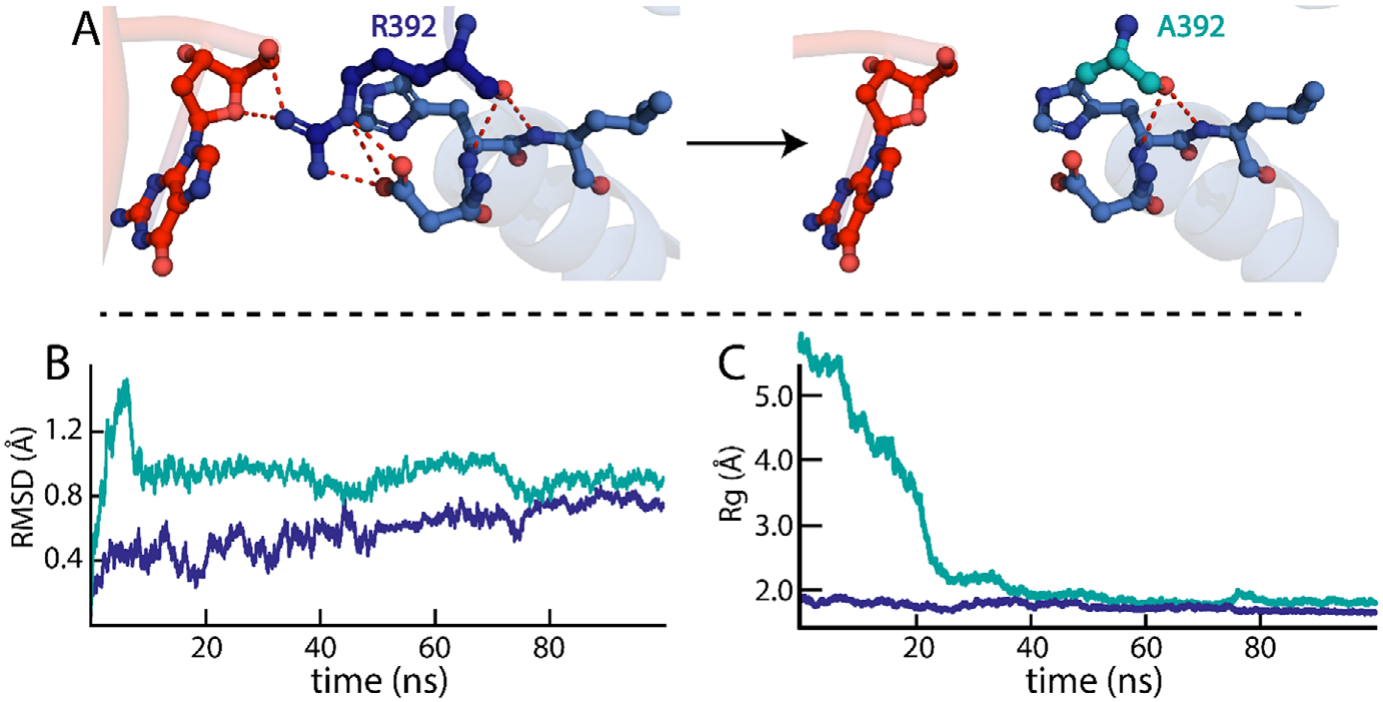
Dynamic stability of the R392A KlF15–DNA complex. (A) demonstrates the loss of the H‐bond when R392 is substituted with alanine. (B) RMSD trajectories of WT (blue) and R392A (teal) KLF15 ZnF domains complexed with DNA. (C) depicts the Rg compactness of WT (blue) and R392A (teal) KLF15 ZnF domains complexed with DNA.

The RMSD analysis of the R392A KLF15 ZnF domain–DNA complex indicated a relatively stable structure (Figure 8B), although the alanine substitution resulted in the loss of five H‐bonds (Figure 8A). During the initial 7 ns of the simulation, the R392A KLF15 ZnF domain–DNA complex RMSD exhibited a significant increase to 1.35 Å before decreasing back to 1.0 Å at 10 ns, suggesting that the R392A KLF15 ZnF domain–DNA complex is undergoing conformational change or rearrangement during the equilibration phase of the simulation. Following this, from 10 to 80 ns, the R392A KLF15 ZnF domain–DNA complex RMSD fluctuated between 0.8 and 1.0 Å, which indicates that the R392A KLF15 ZnF domain–DNA has a more dynamic structure compared to the WT KLF15 ZnF domain–DNA complex. However, in the final phase of the simulation from 80 to 100 ns, the R392A KLF15 ZnF domain–DNA RMSD averaged at 0.9 Å, demonstrating a stable conformation similar to the WT KLF15 ZnF domain–DNA complex. As for the measure of compactness, the Rg trajectories (Figure 7C), the R392A KLF15 ZnF domain–DNA exhibited an initial Rg of 5.5 Å, denoting that the complex starts in a more unfolded conformation. From 0 to 40 ns, the Rg decreased significantly to 2.0 Å indicating the R392A KLF15 ZnF domain–DNA structure is becoming more stable and compact. From 40 ns till the end of the simulation, the R392A KLF15 ZnF domain–DNA complex stabilised with an Rg of 1.9 Å, which is similar to the level of compactness of the WT KLF15 ZnF domain–DNA complex.

In comparison to residues K334 and R344, which substantially impacted the structural stability of the KLF15 ZnF domain–DNA complex, the R392 alanine substitution did not markedly compromise the structural stability of the complex despite the loss of five H‐bonds. When proteins lose specific H‐bonds, they undergo conformational changes to maintain stability by strengthening the salt or increasing the number of hydrophobic interactions. Furthermore, the loss of H‐bonds can result in the rearrangement of water molecules surrounding the protein to compensate for the loss of the H‐bonds [39, 40].

### 3.5 Residue Flexibility Analysis of the KLF ZnF Domain–DNA Complex

**FIGURE 9:**
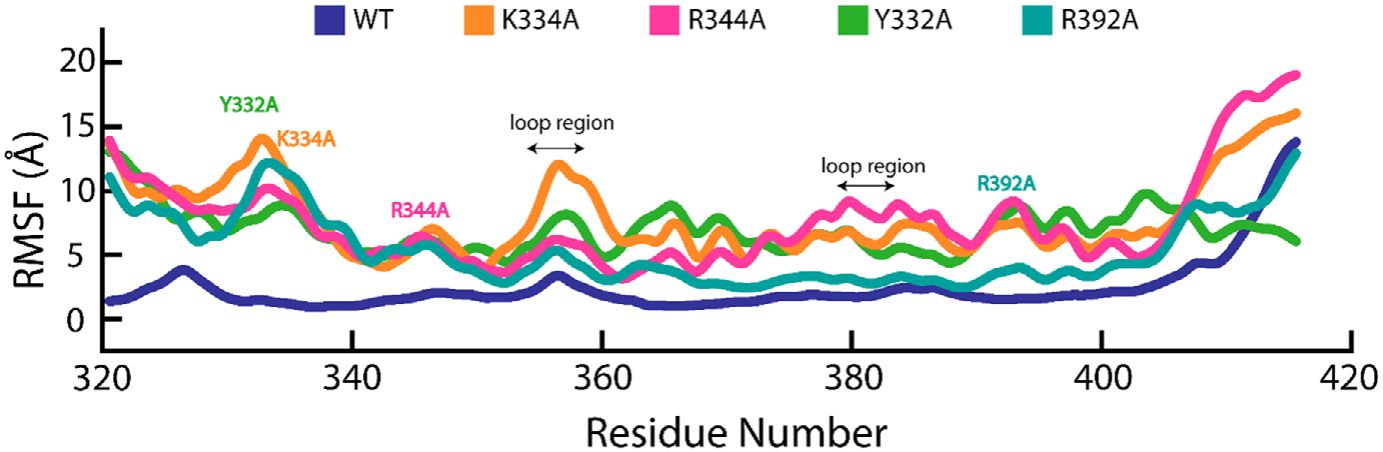
Residue flexibility (RMSF) assessment of the WT (blue), K334A (orange), R344A (magenta) and R392A (teal) mutant KLF15 ZnF domain–DNA complexes.

RMSF measured the flexibility of the residues of KLF15 ZnF domain WT, K334A, R344A, Y332A and R392A bound to DNA by calculating the average deviation from their mean position throughout the 100‐ns simulation (Figure 8). Since RMSF assesses the dynamic behaviour of specific regions, it can determine the optimised conformational states of key KLF15 ZnF residues upon interacting with DNA, with high RMSF values indicating regions of high flexibility and low RMSF values suggesting areas of rigidity (Figure 9).

The WT KLF15 ZnF‐DNA complex exhibits the lowest fluctuations in comparison to the K334A, R344A, Y332A and R392A, suggesting that the structure of the WT KLF15 ZnF domain bound to DNA is stable. The low RMSF values demonstrate a rigid conformation between WT KLF15 ZnF and DNA, which may result from H‐bonds or hydrophobic packing. On the other hand, the K334A KLF15 ZnF–DNA complex demonstrates higher RMSF across the KLF15 ZnF domain region (320–420). The region between residues 320 and 350 experienced the highest flexibility, indicating that the K334 substitution to alanine resulted in the loss of non‐covalent interactions crucial for binding KLF15 ZnF to DNA. In addition, in the loop region between residues 355 and 365, the K334A KLF15 ZnF–DNA complex showed a prominent fluctuation in RMSF, further signifying that the K334 substitution to alanine disrupts the KLF15 ZnF domain binding to DNA.

The RMSF values of R344A KLF15 ZnF–DNA complex are significantly elevated compared to the WT but are slightly lower than K334A. The R344A KLF15 ZnF–DNA complex demonstrated flexibility between residues 320–350, which can result from the loss of H‐bonds between R334 and DNA when substituted with alanine. Similarly to K334A and R344A, the Y332A KLF15 ZnF–DNA complex demonstrated increased RMSF flexibility between residues 320–350. From residue 350 to 410, the Y332A KLF15 ZnF–DNA complex demonstrated a constant RMSF profile flexibility. Notably, KLF15 ZnF domain residues Y332, K334 and R344 are located on the H1 of the KL15 ZnF domain, whereby their subsequent substitution to alanine led to high flexibility in the region between residues 320 and 350, signifying the importance of H1 residues forming the KLF15‐ZnF domain DNA complex.

The R392 residue substitution to alanine positioned on H3 of the KLF15 ZnF domain demonstrated a less flexible complex than the Y332A, K334S and R344A KLF15 ZnF–DNA complexes. The R392A KL15 ZnF–DNA complex demonstrated the highest flexibility between residues 320–350, and from residue 350 to 420, and the R392A KL15 ZnF–DNA complex demonstrated a stable RMSF profile similar to the WT KLF15 ZnF–DNA complex. R392 falls in the loop region of the H3 KL15 ZnF domain structure, where the RMSF flexibility is expected to be highest when substituted with alanine. However, the decrease in mobility, when substituted with alanine, can result from other compensatory interactions or an increase in hydrophobicity with alanine side‐chain interactions [41]. The results demonstrate that K334A, R344A, Y332A and R392A increased the dynamic flexibility of the KLF15 ZnF domain structure and therefore caused more frequent movements of the key regions as witnessed in the Rg results where the compactness or size of the KLF15 ZnF domain was increased.

### 3.6 Binding Free Energy Calculation of KLF15 ZnF‐DNA Complex

The MM/GBSA is robust in calculating the binding free energy of protein–protein, protein–DNA and protein–drug interactions [42]. The MM/GBSA approach is computationally affordable compared to costly alchemical‐free energy methods and offers insight into binding affinities and the impact of amino acid substitution on protein–DNA interactions. To calculate binding energy (ΔG_bind_), simulation trajectories consisting of 20,000 frames were used to estimate the binding free energy of the KLF15 ZnF–DNA complexes WT, Y332A, K334, K334A and R392A (Table 2).

**TABLE 2:** Binding free energy results calculated as MM/GBSA (kcal/mol).

	WT	K334A	R344A	Y332A	R392A
VDWAALS	−102.8 ± 0.13	−34.18 ± 0.36	−47.82 ± 0.35	−51.25 ± 0.45	−24.47 ± 0.21
EEL	−6082.6 ± 1.6	−4163.7 ± 24.6	−4979.0 ± 25.2	−4778.6 ± 24.7	−3878.8 ± 23.1
EGB	6107.2 ± 1.6	4173.3 ± 24.6	4992.3 ± 25.25	4789.6 ± 24.8	3877.3 ± 23.08
ESURF	−15.8 ± 0.02	−5.7 ± 0.06	−8.3 ± 0.06	−7.5 ± 0.06	−4.7633 ± 0.04
DELTA G gas	−6185.4 ± 1.7	−4197.9 ± 24.9	−5026.84 ± 25.5	−4829.8 ± 25.2	−3903.34 ± 23.3
DELTA G solv	6091.4 ± 1.6	4167.5 ± 24.6	4984.1 ± 25.2	4782.1 ± 24.8	3872.5 ± 23.04
Binding Energy (ΔG)	**−94.0 ± 0.17**	**−30.4 ± 0.35**	**−42.8 ± 0.37**	−**47.7 ± 0.42**	**−30.8 ± 0.30**

*Note:* Residue variants and the DeltaG results are highlighted in bold.

The binding affinities analysis demonstrated that the WT KLF15 ZnF domain binds more robustly to DNA than the alanine‐substituted residues K334A, R344A, Y332A and R392A. The WT system exhibited strong van der Waals (vdW) (−102.8 ± 0.13 kcal/mol) and electrostatic (EEL) (−6082.6 ± 1.6 kcal/mol) interactions. In contrast, K334A and R392A showed a significant reduction in vdW and EEL, with K334A demonstrating a vdW of (−34.18 ± 0.36 kcal/mol) and an EEL (−4163.7 ± 24.6 kcal/mol), whereby R392A showed a vdW of (−24.47 ± 0.21 kcal/mol) and EEL (−3878.8 ± 23.1 kcal/mol). This may indicate that K334 and R392 contribute to hydrophobic and electrostatic interactions between the KL15 Znf domain and DNA. On the other hand, the R344A and Y332A demonstrated a less elaborate reduction in vdW and EEL, with R344A having a vdW of (−47.82 ± 0.35 kcal/mol) and an EEL (−4979 ± 25.2 kcal/mol), whereas Y332A presented a vdW of (−51.25 ± 0.45 kcal/mol) and EEL (−4778.6 ± 24.7 kcal/mol). This could indicate that R344 and Y332 contribute primarily to polar interactions between the KL15 ZnF domain and DNA. Nonpolar solvation effects (Esurf) are less impactful overall but exhibit notable differences among variants; WT has a favourable Esurf (−15.8 ± 0.02 kcal/mol), while K334A (+5.7 ± 0.06 kcal/mol) shows a positive contribution, further reducing its binding affinity. The polar solvation energy EGB of the WT complex (+6107 kcal/mol) offsets its electrostatic contribution. The K334A and R392A KLF15 ZnF show a less favourable EGB, displaying the weakest contributions.

The binding free energy (ΔG_bind_) is the final estimated free energy of the KLF15 ZnF–DNA complex, calculated from the sum of the gas phase energy (DELTA G gas) and solvation energy (DELTA G solv; Table 2). The WT KLF15 ZnF domain exhibited the most favourable ΔG_bind_ to DNA (−94.0 ± 0.17 kcal/mol) compared to K334A (−30.4 ± 0.35 kcal/mol), R344A (−42.8 ± 0.37 kcal/mol), (−47.7 ± 0.42 kcal/mol) and R392A (−30.8 ± 0.30 kcal/mol), with K334A and R392A demonstrating the weakest binding affinities, indicating the critical role of K334 and R392 for the KLF15 ZnF domain binding to DNA. Free binding energy provided insight into the strength and stability of molecular interactions and thermodynamic favourability between the KLF15 ZnF domain and DNA and showed that both residues K334 and R392 are critical for complex formation between KLF15 ZnF and DNA. Alanine substitution significantly altered K334A and R392A binding affinities. However, the alanine substitution docking analysis (Table 1) and RMSD, Rg and RMSF results showed that K334A affected the stability of the KLF15 ZnF‐DNA complex, which fits with the K334Aweaker ΔG_bind_ (−30.4 ± 0.35 kcal/mol). In contrast, the R392A RMSD, Rg and RMSF demonstrated a more stable structure than the other variants; as such, the weaker ΔG_bind_ (−30.8 ± 0.30 kcal/mol) could be the result of R392 positioned in the H3 c‐terminus of the ZnF binding domain, which is critical for forming the KL15 ZnF–DNA complex. Therefore, the alanine substitution might not have affected the KLF15 ZnF domain DNA complex dynamics of R392A KL15 ZnF binding to DNA, but the alanine substitution possibly affected the H‐bonding and polar interactions with DNA.

## 4 Conclusion

KLF15 is a transcription factor that binds to DNA through the ZnF domain and plays a significant role in various physiological processes, including gluconeogenesis, lipid metabolism and the regulation of blood sugar levels [17]. As such, the KLF15 ZnF domain is a compelling target for therapeutic interventions in metabolic syndromes, especially T2DM. This study used advanced computational biology tools to outline the interaction mechanism between the KLF15 ZnF domain and DNA. The KLF15 ZnF domain structure has not been elucidated experimentally; therefore, AlphaFold 3.0 was utilised to model the KLF15 ZnF domain complex with DNA. Furthermore, alanine scanning and MD simulations characterised the interaction mechanism between the KLF15 ZnF domain and DNA. We identified four residues, namely K334, R344, Y332 and R392, which are crucial for binding and recognising DNA and may be critical for the transcriptional regulation of genes involved in gluconeogenesis and blood sugar level maintenance.

In particular, residues K334 and R392, positioned on H1 and H3 of the KLF15 ZnF domain, are critical for complex formation between KLF15 ZnF and DNA. Alanine substitution significantly altered K334A and R392 and affected hydrophobic and electrostatic interactions, which led to reduced binding energies of KLF15 ZnF to DNA. This underscores their pivotal role in facilitating the recognition and binding of the KLF15 ZnF domain and indicates their potential as targets in designing novel therapeutic agents for T2DM and its complications.

## Author Contributions

**Anwar Mohammad:** conceptualization (lead), investigation (lead), methodology (lead), writing – original draft (lead). **Jehad Abubaker:** formal analysis (lead), writing – original draft (equal), writing – review and editing (lead). **Sulaiman K. Marafie:** formal analysis (supporting), methodology (supporting). **Eman AlShawaf:** formal analysis (supporting), methodology (supporting). **Hamad Ali:** investigation (equal), writing – original draft (equal). **Fahd Al‐Mulla:** conceptualization (lead), writing – original draft (lead).

## Conflicts of Interest

The authors declare no conflicts of interest.

## Data Availability

Data available on request from the authors.
